# Synthesis, Superoxide Dismutase Mimetic and Anticancer Activities of Metal Complexes of 2,2-Dimethylpentanedioic Acid(2dmepdaH_2_) and 3,3-Dimethylpentanedioic acid(3dmepdaH_2_): X-Ray Crystal Structures of [Cu(3dmepda)(bipy)]_2_· 6H_2_O and [Cu(2dmepda)(bipy)(EtOH)]_2_· 4EtOH (bipy = 2,2′Bipyridine)

**DOI:** 10.1155/BCA/2006/80283

**Published:** 2006-03-20

**Authors:** Michael Devereux, Malachy McCann, Denis O'Shea, Mark O'Connor, Eileen Kiely, Vickie McKee, Declan Naughton, Anna Fisher, Andrew Kellett, Maureen Walsh, Denise Egan, Carol Deegan

**Affiliations:** ^1^Dublin Institute of Technology, Cathal Brugha Street, Dublin 1, Ireland; ^2^Department of Chemistry, National University of Ireland Maynooth Maynooth, Co Kildare, Ireland; ^3^Department of Chemistry, Loughborough University, Loughborough, Leicestershire, LE11 3TU, UK; ^4^School of Pharmacy and Biomolecular Sciences, University of Brighton, Cockcroft Building, Moulsecoomb, Brighton BN2 4GJ, UK; ^5^Department of Applied Science, Institute of Technology, Tallaght, Dublin 24, Ireland

## Abstract

2,2-dimethylpentanedioic acid (2dmepdaH_2_) and 3,3-dimethylpentanedioic acid (3dmepdaH_2_) reacted with copper(II) acetate to give [Cu(2dmepda)(H_2_O)_3_]_2_ (**1**) and [Cu(3dmepda)(H_2_O)_3_]_2_ (**2**). Reaction of (**1**) and (**2**) with 1,10-phenanthroline and 2,2′-bipyridine yielded [Cu(2dmepda)(phen)(H_2_O)]_2_0.5phen (**3**), [Cu(2dmepda)(bipy)(H_2_O)]_2_ (**4**), [Cu(2dmepda)(bipy)(EtOH)]_2_· 2EtOH (**4A**), [Cu(3dmepda)(phen)(H_2_O)]_2_ (**5**), and [Cu(3dmepda)(bipy)(H_2_O)]_2_· (**6**). The structures of (**4A**) and (**6**) each consists of a [Cu(bipy)(dicarboxylate)(solvent)]_2_ dimer. The superoxide dismutase (SOD) mimetic activity of the novel copper complexes and their manganese analogues was investigated. The dimethyl sulphoxide(DMSO) soluble complexes (**1**)–(**4**) and (**6**) were assessed for their cancer chemotherapeutic potential towards hepatocellular carcinoma and kidney adenocarcinoma cell lines. The 1,10-phenanthroline containing complex [Cu(2dmepda)(phen)(H_2_O)]_2_0.5phen (**3**) was the most potent with activity that compares well to that of *cis*platin.

## INTRODUCTION

A range of monocarboxylic acids are known to have a variety of pharmacological effects. Salicylic acid and its derivatives, for example, have been shown to possess anti-inflammatory and antitumour activity [1]. Upon coordination to a suitable metal centre, the biologically active carboxylic acids often become more effective and desirable drugs [2]. The carboxylate group is an important class of ligand in inorganic and bioinorganic chemistry, metal complexes containing monocarboxylic acids are well known, and the publication of many structurally characterised examples of this class of compound has demonstrated its versatility as an inner-sphere ligand [3]. The coordination chemistry of dicarboxylic acids is far less developed as polymeric products are usually obtained, and indeed structural information for this class of complex is relatively scarce. Recently, we have shown that the reaction of polymeric dicarboxylate complexes of copper(II), manganese(II), and cobalt(II) with the N,N-donor ligands 1,10-phenanthroline and 2,2′-bipyridine can lead to the synthesis of crystalline compounds which are easily structurally characterised by X-ray methods [4–9]. A number of the manganese complexes have been found to exhibit excellent catalase-mimetic activity [5,8] and it has also been demonstrated that N-donor derivatives of the dicarboxylate complexes of a range of metals are very effective antifungal agents possessing significantly different modes of action to the state-of-the-art prescription drugs [10–12].

As part of our ongoing studies into the synthesis, biomimetic, and biological applications of novel transition metal carboxylate complexes, we report here the properties and structures for copper(II) complexes of 2,2-dimethylpentanedioic acid and 3,3-dimethylpentanedioic acid. The ability of the copper complexes to dismutate superoxide has been compared to manganese(II) analogues we have previously published [9]. The chemotherapeutic potentials of several of the novel copper complexes towards two tumourigenic model cell lines are also discussed.

## EXPERIMENTS

### Chemistry

Chemicals were purchased from commercial sources and used without further purification. IR spectra were recorded in the region 4000–400 cm^−1^ on a Nicolet-400 Impact spectrometer. Magnetic susceptibility measurements were made using a Johnson Matthey magnetic susceptibility balance. [HgCo(SCN)_4_] was used as a reference. Satisfactory microanalytical data for the complexes were reported by the Microanalytical Laboratory, University College Cork, Ireland. The manganese complexes were synthesised using a method previously published [9].

[Cu(2dmepda)(H_2_O)_3_]_2_ (**1**)

To a solution of 2,2-dimethylpentanedioic acid (2dmepdaH_2_) (1.615 g, 0.01 moles) in H_2_O (100 cm^3^) was added [Cu_2_(CH_3_CO_2_)_4_· 2H_2_O] (4.05 g , 0.001 moles) and the resulting green mixture was refluxed for 2 hours. Upon cooling, the green solid which deposited was filtered off, washed with two portions of distilled H_2_O, and dried in vacuo. Yield: 2.21 g (82.86%); calc %: C 30.47 H 5.85; found %: C 30.94 H 5.73; IR(KBr): 3423, 2963, 2919, 1597, 1477, 1421, 1373, 1305, 1241, 1173, 1148, 1053, 824, 784, 724, 684, 636, 462 cm^−1^. *μ*_eff_ = 1.86 BM; solubility: insoluble in water, soluble in ethanol, and partially soluble in methanol and acetone.

[Cu(3dmepda)(H_2_O)_3_]_2_ (**2**)

To a solution of 3,3-dimethylpentanedioic acid (3dmepdaH_2_) (1.60, 0.01 moles) in distilled H_2_O (100 cm^3^) was added [Cu_2_(CH_3_CO_2_)_4_ · 2H_2_O] (4.05 g, 0.01 moles) and the resulting green mixture was refluxed for 2 hours. Upon cooling, the green solid which deposited was filtered off, washed with two portions of distilled H_2_O, and dried in vacuo. Yield: 1.88 g (75.91%); calc %: C 30.49 H 4.85; found %: C 30.82 H 5.10; IR(KBr): 3652, 3423, 2951, 1605, 1405, 1317, 1277, 1248, 1176, 1161, 904, 804, 768, 732, 676, 624, 468 cm^−1^. *μ*_eff_ = 1.61 BM; solubility: insoluble in water and acetone and partially soluble in heated ethanol and methanol.

[Cu(2dmepda)(phen)(H_2_O)]_2_· 0.5phen (**3**)

To a solution of [Cu(2dmepda)(H_2_O)_3_]_2_ (**1**) (0.75 g, 0.0015 moles), in ethanol (50 cm^3^), was added 1,10-phenanthroline (2.48 g, 0.0136 moles) and the mixture was refluxed for two hours. The resulting dark green solution was concentrated to approximately 5 cm^3^ and acetone (10 cm^3^) was added and after several days at room temperature, a blue solid precipitated. The product was filtered off, washed with a small volume of ethanol, and then dried in vacuo. Yield: 1.02 g (76.66%); calc %: C 58.87 H 4.74, N 8.24; found %: C 58.55 H 4.66 N 8.10; IR(KBr): 3399, 3055, 2959, 2915, 2859, 1561, 1517, 1469, 1425, 1397, 1365, 1297, 1225, 1145, 1105, 857, 720, 648 cm^−1^. *μ*_eff_ = 1.92 BM; solubility: soluble in water, ethanol and methanol . Insoluble in acetone.

[Cu(2dmepda)(bipy)(H_2_O)]_2_ (**4**)

To a solution of [Cu(2dmepda)(H_2_O)_3_]_2_ (**1**) (0.75 g, 0.0015 moles), in ethanol (50 cm^3^) was added 2,2′-bipyridine (2.12 g, 0.0136 moles) and the resulting dark green solution was refluxed for 2 hours. Upon standing for several days at room temperature, a blue powder precipitated, was filtered, washed with a small portion of methanol and acetone, and then placed in an oven to dry. Yield: 0.98 g (72.90%); calc %: C 51.57 H 5.09 N 7.08; found %: C 51.07 H 4.93 N 7.07; IR: 3451, 3111, 3079, 2963, 2923, 2875, 1569, 1477, 1445, 1401, 1365, 1309, 1237, 1157, 1057, 1034, 905, 836, 776, 728, 640 cm^−1^. *μ*_eff_ = 2.12; solubility: insoluble in water, ethanol and acetone, soluble in methanol.

Upon standing for several weeks, the filtrate yielded a small quantity of crystals of [Cu(2dmepda)(bipy)(EtOH)]_2_· 2EtOH (**4A**) which were suitable for X-ray analysis.

[Cu(3dmepda)(phen)(H_2_O)]_2_ (**5**)

To a solution of [Cu(3dmepda)(H_2_O)_3_]_2_ (**2**) (0.52 g, .00103 moles) in ethanol (50 cm^3^) was added 1,10–phenanthroline (1.64 g, 0.0092 moles) and the resulting dark brown solution was refluxed for two hours. The solution was then cooled and concentrated to 5 cm^3^ and acetone (15 cm^3^) was added precipitating a green solid which was filtered off, washed with a small volume of ethanol, and dried in vacuo. Yield: 0.22 g (30.91%); calc %: C 58.87 H 4.74 N 8.24; found %: C 58.96 H 4.56 N 8.94%; IR(KBr):3395, 1557, 1517, 1425, 1381, 1305, 1253, 1225, 1145, 1105, 849,776, 724 cm^−1^. *μ*_eff_ = 1.98; solubility: insoluble in water, ethanol, and acetone. Soluble in methanol.

[Cu(3dmepda)(bipy)(H_2_O)]_2_· (**6**)

To a solution of [Cu(3dmepda)(H_2_O)_3_]_2_ (**2**) (0.51 g, 0.00101 moles) in ethanol (50 cm^3^), was added 2,2′-bipyridine (1.44 g, 0.0092 moles) and the resulting dark brown solution was refluxed for 2 hours. The solution was then cooled and concentrated to 5 cm^3^ and acetone (15 cm^3^) was added precipitating a blue solid which was filtered off, washed with a small volume of ethanol, and dried in vacuo. Yield: 0.21 g (32.34%); calc %: C 51.57 H 5.09 N 7.08; found %: C 52.33 H 4.87 N 7.27; IR(KBr): IR: 3399, 3055, 2959, 2915, 2859, 1561, 1517, 1469, 1425, 1397, 1365, 1297, 1225, 1145, 1105, 857, 720, 648 cm^−1^· *μ*_eff_ = 2.22; solubility: insoluble in water, ethanol, and acetone.

Upon standing for several weeks, the filtrate yielded a small quantity of crystals of [Cu(3dmepda)(bipy)(H_2_O)]_2_· 6H_2_O which were suitable for X-ray analysis. 

### X-ray Crystallography

Both data sets were collected at 153(2) K using a Siemens P4 diffractometer with Mo-K_*α*_ radiation (*λ* = 0.71073 Å, 2*θ*_max_ = 25.0°) and corrected for Lorentz, polarisation, and absorption effects. The structure for (**4A**) was solved by direct methods and (**6**) was solved using a Patterson map. Both structures were refined by full-matrix least-squares on F^2^, using all the reflections; the non-hydrogen atoms were refined with anisotropic atomic displacement parameters and hydrogen atoms bonded to carbon atoms were inserted at calculated positions. Hydrogen atoms bonded to oxygen were located from difference Fourier maps and not further refined. There were no significant residual peaks in either electron density map. Details of the collection and refinement are given in Table 1. All programs used in the structure solution and refinement are contained in the SHELXTL package [13].

#### Superoxide dismutase activity

The O_2_^•−^ dismutase activities of the metal complexes were assessed using a modified NBT assay with xanthine-xanthine oxidase system as the source of O_2_^•−^ [14]. All reagents were obtained from Sigma-Aldrich Chemical Co Ltd and assays were run in 3 mL of solution. Results are graphed as the inhibition % of NBT reduction for three concentrations. Tabulated results were derived from linear regression analyses and are given as the concentration (*μ*M) equivalent to 1 U bovine erythrocyte superoxide dismutase (SOD).

#### Cytotoxiciy testing

Dimethyl sulphoxide (DMSO) and all cell culture reagents and media were purchased from Sigma-Aldrich Ireland, Ltd, unless otherwise stated.

Cytotoxicity assays were performed using two human malignant model cell lines in order to assess the cancer chemotherapeutic potential of the Cu complexes of 2,2- and 3,3-dimethylpentanedioic acid. Hepatocellular carcinoma (HepG2) and kidney adenocarcinoma (A-496) cell lines were purchased from the ATCC. All cell lines were grown as monolayers in Eagle's minimum essential medium, supplemented with 2 mM L-glutamine and Earle's balanced salt solution, containing 1.5 g dm^−3^ sodium bicarbonate, 0.1 mM nonessential amino acids, 1.0 mM sodium pyruvate, 100 U cm^−3^ penicillin, and 100 *μ*g cm^−3^ streptomycin supplemented to contain 10% (v/v) foetal bovine serum (Flow laboratories, Herts, UK). All cells were grown at 37^°^C in a humidified atmosphere, in the presence of 5% CO_2_ and were in the exponential phase of growth at the time of assay. Cytotoxicity was assessed using MTT assay. Each of the two cell lines (100 *μ*L) were seeded at a density of 5×10^4^ cells cm^−3^ into sterile 96 well flat-bottomed plates (Falcon Plastics, Becton Dickinson) and grown in 5% CO_2_ at 37^°^C. Test compounds were dissolved in DMSO and diluted with culture media. The maximum percentage of DMSO present in all wells was 0.2% (v/v). Each drug solution (100 *μ*L) was added to replicate wells in the concentration range of 0.1–1000 *μ*M and incubated for 96 hours. A miniaturised viability assay using 3-(4,5-dimethylthiazol-2-yl)-2,5-diphenyl tetrazolium bromide (MTT) was carried out according to the method described by Mosmann [15]. The IC_50_value, defined as the drug concentration causing a 50% reduction in cellular viability, was calculated for each drug. Each assay was carried out using five replicates and repeated on at least three separate occasions. Viability was calculated as a percentage of solvent-treated control cells, and expressed as a percentage of the control. The significance of any reduction in cellular viability was determined using one-way ANOVA (analysis of variance). A probability of 0.05 or less was deemed statistically significant.

### RESULTS AND DISCUSSION

**Scheme 1:**
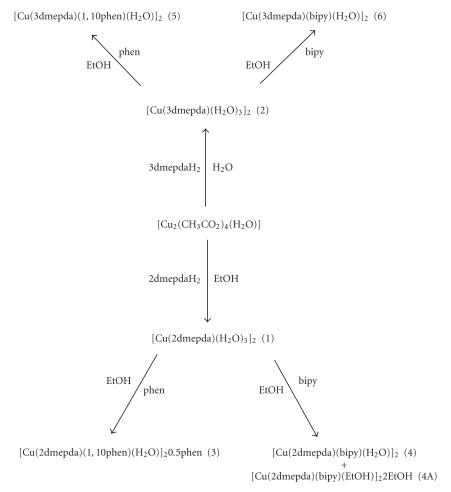
Synthetic routes to the complexes (1)–(6).

Synthetic routes to the complexes (**1**)–(**6**) are shown in Scheme 1. Reaction of either 2,2-dimethylpentanedioic acid (2dmepdaH_2_) or 3,3-dimethylpentanedioic acid (3dmepdaH_2_) with copper(II) acetate gave the complexes [Cu(2-dmepda)(H_2_O)_3_]_2_ (**1**) and [Cu(2-dmepda)(H_2_O)_3_]_2_ (**2**), respectively. Reaction of (**1**) with either 1,10-phenanthroline or 2,2′-bipyridine resulted in the synthesis of [Cu(2dmepda)(phen)(H_2_O)]_2_· 0.5phen (**3**), [Cu(2dmepda)(bipy)(H_2_O)]_2_ (**4**), and [Cu(2dmepda)(bipy)(EtOH)]_2_· 2EtOH (**4A**). [Cu(3dmepda)(phen)(H_2_O)]_2_ (**5**) and [Cu(3dmepda)(bipy)(H_2_O)]_2_· (**6**) were obtained when (**2**) was treated similarly.

Crystals of the hepta hydrate of (**6**) {[Cu(3dmepda)(bipy)(H_2_O)]_2_· 6H_2_O} were obtained from the mother liquor by slow evaporation.

**Figure 1:**
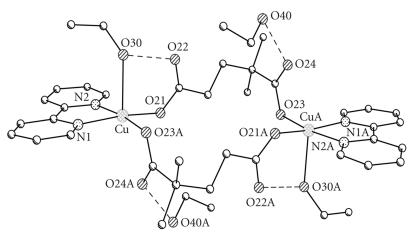
The structure of dimeric [Cu(2dmepda)(bipy)(EtOH)]_2_ · 2EtOH (**4A**).

**Figure 2:**
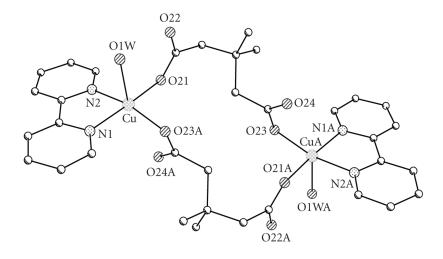
The dimeric structure of [Cu(3dmepda)(bipy)(H_2_O)]_2_ · (**6**) (crystals of (**6**) were obtained as the hepta hydrate. The six water molecules of crystalisation have been omitted for clarity).

The X-ray crystal structures of [Cu(2dmepda)(bipy)(EtOH)]_2_· 2EtOH (**4A**) and [Cu(3dmepda)(bipy)(H_2_O)]_2_· 6H_2_O (**6**) are shown in Figures 1 and 2 and selected bond lengths and angles are listed in Tables 2 and 3, respectively.

**Figure 3:**
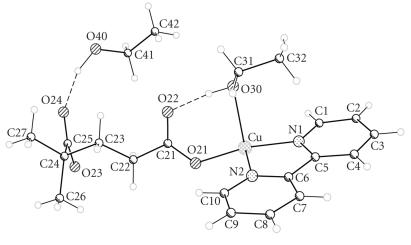
The hydrogen bonding in [Cu(2dmepda)(bipy)(EtOH)]_2_ · 2EtOH (**4A**).

**Figure 4:**
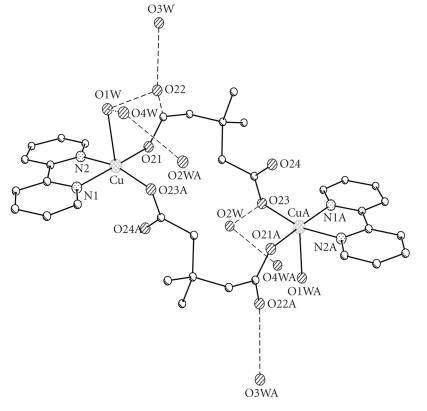
The hydrogen bonding in [Cu(3dmepda)(bipy)(H_2_O)]_2_ · (**6**).

The two structures are closely related, each consisting of a [Cu(bipy)(dicarboxylate)(solvent)]_2_ dimer located on a centre of symmetry. The copper ions are five-coordinate and have approximately square pyramidal geometry with the coordinated solvent as the apical donor (ethanol and water for (**4A**) and (**6**), resp). The donors in the basal plane comprise the two bipyridine nitrogen atoms and two carboxylate oxygen atoms, one from each of two dicarboxylate ligands. The apical ligand forms a hydrogen bond to the uncoordinated oxygen of one of the dicarboxylate groups {O(30)−O(22) 2.595(3) Å and O(1w)−O(22) 2.644(3) Å in (**4A**) and (**6**), resp} (Figures 3 and 4). The two copper ions in the dimeric unit are therefore linked by two dicarboxylate groups.

**Figure 5:**
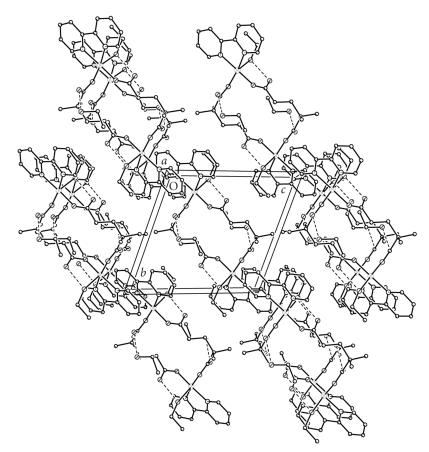
The packing diagram for [Cu(2dmepda)(bipy)(EtOH)]_2_ · 2EtOH (**4A**) showing the π-π stacking.

The uncoordinated ethanol molecule in (**4A**) is hydrogen-bonded to the second uncoordinated carboxylate oxygen {O(40)−O(24) 2.808(3) Å} (Figure 3); there are no other hydrogen atoms available for further hydrogen bonding. The molecules are linked into chains by *π*-*π* bonding between the bipyridine ligands (Figure 5) on neighbouring molecules on the opposite side of the copper to the coordinated ethanol molecule (interplanar distance approximately 3.6 Å). Interaction on the opposite plane is prevented by the apical ligand.

**Figure 6:**
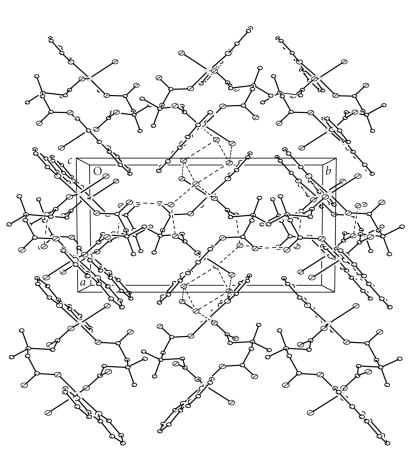
The packing diagram for [Cu(3dmepda)(bipy)(H_2_O)]_2_ · 6H_2_O (**6**) showing the π-π stacking.

There are three uncoordinated water molecules in the asymmetric unit of (**6**) (six per dimer) and these are all involved in hydrogen bonding to the carboxylates, the coordinated water, and each other (Figure 6). These hydrogen bonds extend through the lattice and there is also some weak *π*-*π* interaction between each bipyridine ring and one neighbouring ring from another dimmer on the same side as the coordinated water molecule (interplanar distance ca 3.6 Å).

As was the case for (**4A**) and (**6**), the IR spectra of complexes (**1**)–(**3**) and (**5**) all contain prominent *ν*_asym_(COO) stretching bands in the region 1550–1610 cm^−1^ and *ν*_asym_(COO) stretching bands in the region 1400–1390 cm^−1^ [Δ*ν*(COO) = 164–200 cm^−1^]. The Δ*ν*(COO) values suggest that the coordination modes of the dicarboxylate ligands in these complexes may be similar to those found in (**4A**) and (**6**) [16].

The room-temperature magnetic moments of powdered samples of complexes (**1**)–(**6**) (μ_eff_ = 1.61–2.22 BM) are consistent for copper(II) complexes where there are no significant exchange interactions between adjacent metal centres [17]. As was the case for (**4A**) and (**6**) complexes, (**1**)–(**3**) and (**5**) were found to be quite soluble suggesting that they are not polymeric supporting their formulation as dimeric complexes. The inclusion of 0.5 molecules of phen of crystallisation in (**3**) is not unusual as complexes incorporating similar ligands as uncoordinated molecules have been previously structurally characterised and reported by this group[18].

### SOD activity

Since the discovery of the functionality of the enzyme SOD [19], intensive efforts have been made to develop the enzyme as a therapeutic agent for the treatment of diseases such as rheumatoid arthritis and osteoarthritis, conditions which are associated with oxidative stress [20]. For several reasons, such as the size and instability of the SOD enzyme, these attempts to develop the natural enzyme for clinical use have been largely unsuccessful. A great deal of of interest has been shown in the development of therapeutic SOD mimetics for the scavenging of superoxide(O_2_^•−^) which is a precursor to reactive oxygen and nitrogen species(RONS) known to contribute to oxidative stress [21].

**Figure 7:**
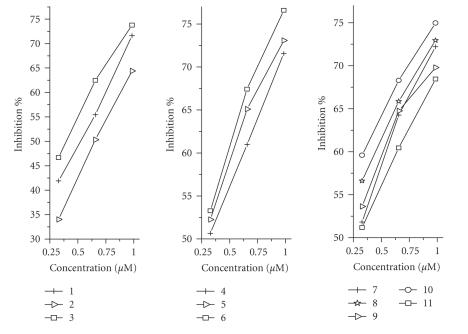
SOD activity profiles of compounds (**1**)–(**11**) as assessed by the NBT assay.

Recently, Cu(II) and Mn(II) complexes of the polycarboxylate EDTA and related chelators have been shown to exhibit significant SOD mimetic activities (using a modified nitrobluetetrazolium (NBT) assay) [22,23]. The SOD mimetic activities of the copper(II) complexes (**1**)–(**6**) and five of their manganese(II) analogues (complexes (**7**)–(**11**)—previously published [9]) were determined. The results are given in Figure 7 and Table 4 as concentrations equivalent to one unit of bovine erythrocyte SOD. Significant activities were seen for all compounds tested with one unit of SOD activity arising from the range of 0.37 to 0.96 *μ*M aqueous solutions. The copper complexes (**1**)–(**3**) and (**5**)–(**6**) are the most active comparing favourably with a number of synthetic SOD mimics developed for therapeutic purposes [21].

### Anticancer studies

**Figure 8:**
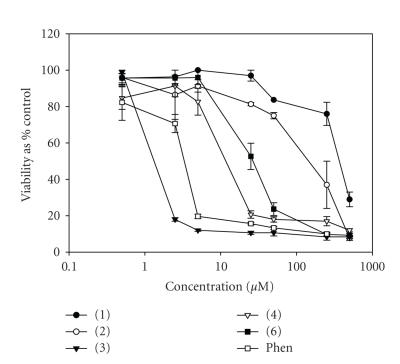
Effects of (**1**)–(**4**), (**6**), and phen on the viability of HepG2 cells (human hepatocellular), following continuous incubation for 96 hours, with increasing drug concentration (0.1–500 *μ*M). Bars indicate standard error of the mean (SEM) and results were statistically significant from control at P < .05. Results are representative of three independent experiments (*n* = 3).

**Figure 9:**
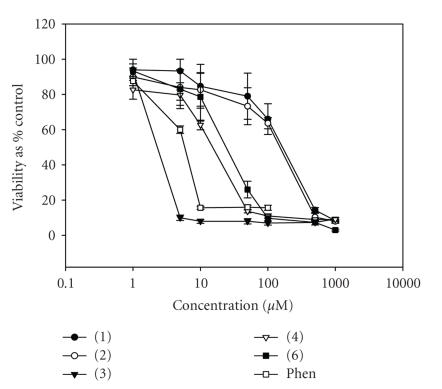
(1)–(4), (6), and phen on the viability of A-498 cells (human renal cell carcinoma), following continuous incubation for 96 hours, with increasing drug concentration (0.1−500 *μ*M). Bars indicate standard error of the mean (SEM) and results were statistically significant from control at *P* < .05. Results are representative of three independent experiments (*n* = 3).

We have recently shown that transition metal complexes of selected carboxylate and dicarboxylate ligands exhibit significant in vitro anticancer activity [12,24]. The ability of the DMSO soluble copper complexes (**1**)–(**4**) and (**6**) to kill human-derived cancer cells was investigated using HepG2 and A-498 cells and a standard bioassay, MTT. Cells were continuously exposed to test agent for 96 hours, and their effects on cellular viability was evaluated. It was intended that the results from these studies would allow the identification of those derivatives with cancer chemotherapeutic potential. Therefore, profiles of cell viability against complex concentration were established (Figures 8 and 9) and were used to calculate the IC_50_ values for each derivative (Table 5). Comparison of IC_50_values allowed the relative potency of each of the test compounds to be determined and ranked.

All five compounds screened displayed a concentration-dependent cytotoxic profile across the two cell lines studied here. The order of the observed cytotoxicity was seen as (**3**) > (**4**) > (**6**) > (**2**) > (**1**), with (**3**) appearing as the most potent and (**1**) as the least potent. The results presented in Table 5 also illustrate that the free Cu salt was incapable of eliciting a cytotoxic response. The inclusion of the N,N-donor ligands 1,10-phenanthroline(phen) and 2,2′-bipyridine(bipy) in the simple Cu(II) complexes of the 2,2- and 3,3-dimethylpentanedioic acids significantly increased the potency of the system. However, it is also noteworthy that the metal-free phenanthroline is itself significantly cytotoxic and that the best copper complex containing it (**3**) is approximately 3 and 2 times more potent for the respective cell lines. Complex (**3**) was capable of killing both cancer-derived cell lines at very low concentrations with IC_50_ values of 1.70 and 1.55 *μ*M (equivalent to 1.49 and 1.37 *μ*g/mL), for the liver and kidney cell lines, respectively. The activity of (**3**) falls well within the accepted activity parameters adopted for in vitro screening of potential chemotherapeutic drugs [25]. Furthermore, the IC_50_ values for (**3**) are comparable to those of the clinically used drug cis platin [26]. The relatively unique structure found in this class of compound may serve to provide a lead structure for the development of further compounds with an even greater cancer chemotherapeutic potential.

## SUPPLEMENTARY MATERIAL

Crystallographic data have been deposited with the CCDC (12 Union Road, Cambridge, CB2 1EZ, UK) and are available on request quoting the deposition numbers CCDC238512 and CCDC238511, respectively.

## Figures and Tables

**Table 1:** Crystal data and structure refinement for [Cu(2dmepda)(bipy)(EtOH)]_2_ · 2EtOH (**4A**) and [Cu(3dmepda)(bipy)(H_2_O)]_2_ · 6H_2_O (**6**).

Empirical formula	C_34_H_54_Cu_2_N_4_O_16_	C_42_H_60_Cu_2_N_4_O_12_
Formula weight	449.94	470.01
Temperature	153(2) K	153(2) K
Wavelength	0.71073 Å	0.71073 Å
Crystal system	Monoclinic	Triclinic
Space group	P2(1)/c	P-1
Unit-cell dimensions	a *=*11.083(3) Å; *α* *=*90°	a *=*9.2092(17) Å; *α* *=*106.374(12)°
	b *=*15.343(4) Å; *β* *=*96.85(2)°	b *=*11.046(2) Å; *β* *=*105.160(13)°
	c *=*12.195(3) Å; *γ* *=*90°	c *=*12.2098(18) Å; *γ* *=*101.568(13)°
Volume	2058.9(10) Å^3^	1098.6(3) Å^3^
Z	2	1
Density (calculated)	1.452 Mg/m^3^	1.421 Mg/m^3^
Absorption coefficient	1.106 mm^−1^	1.033 mm^−1^
F(000)	940	494
Crystal size	0.32 × 0.42 × 0.32 mm^3^	0.50 × 0.46 × 0.36 mm^3^
Theta range for data collection	2.14 to 25.00°	2.01 to 25.00°
Index ranges	0 ≤ h ≤ 13, −18 ≤ k ≤ 1, −14 ≤ l ≤ 14	−10 ≤ h ≤ 0, −12 ≤ k ≤ 12, −14 ≤ l ≤ 14
Reflections collected	4145	4044
Independent reflections	3633 [R(int) *=*0.0192]	3780 [R(int) *=*0.0127]
Completeness to theta *=*25.00°	99.9%	97.8%
Absorption correction	Empirical	Empirical
Max and min transmission	0.7975 and 0.7547	0.8507 and 0.6635
Refinement method	Full-matrix least-squares on F^2^	Full-matrix least-squares on F^2^
Data/restraints/parameters	363/0/253	3780/0/271
Goodness-of-fit on F^2^	1.025	1.072
Final R indices [I > 2sigma(I)]	R1 *=*0.0365, wR2 *=*0.0719	R1 *=*0.0311, wR2 *=*0.0733
R indices (all data)	R1 *=*0.0608, wR2 *=*0.0795	R1 *=*0.0387, wR2 *=*0.0764
Largest diff peak and hole	0.292 and −0.309 e·Å^−3^	0.526 and −0.314 e·Å^−3^

**Table 2:** Bond lengths (Å ) and angles (∘) for [Cu(2dmepda)(bipy)(EtOH)]_2_ · 2EtOH (**4A**).

Cu−O(21)	1.955(2)
Cu−O(23*)	1.9836(19)
Cu−N(1)	2.010(2)
Cu−N(2)	2.003(2)
Cu−O(1W)	2.307(2)
O(21)−Cu−O(23*)	90.22(8)
O(21)−Cu−N(2)	92.99(9)
O(23*)−Cu−N(2)	165.11(9)
O(21)−Cu−N(1)	173.23(9)
O(23*)−Cu−N(1)	95.09(9)
N(2)−Cu−N(1)	80.82(10)
O(21)−Cu−O(1W)	95.60(8)
O(23*)−Cu−O(1W)	99.71(8)
N(2)−Cu−O(1W)	94.45(9)
N(1)−Cu−O(1W)	87.66(8)

Symmetry transformations used to generate equivalent atoms: *3 − *x*, − *y*, 2 − *z*, ^†^1 − *x*, 1 − *y*, 1 − *z*.

**Table 3:** Bond lengths (Å ) and angles (∘) for [Cu(3dmepda)(bipy)(H_2_O)]_2_ · 6H_2_O (**6**).

Cu−O(21)	1.9376(17)
Cu−O(23^†^)	1.9452(16)
Cu−N(1)	2.0116(19)
Cu−N(2)	2.0165(19)
Cu−O(30)	2.4119(18)
O(21)−Cu−O(23^†^)	92.09(7)
O(21)−Cu−N(1)	169.65(7)
O(23^†^)−Cu−N(1)	93.50(7)
O(21)−Cu−N(2)	93.31(8)
O(23^†^)−Cu−N(2)	172.23(7)
N(1)−Cu−N(2)	80.34(8)
O(21)−Cu−O(30)	97.17(7)
O(23^†^)−Cu−O(30)	90.13(7)
N(1)−Cu−O(30)	91.52(7)
N(2)−Cu−O(30)	94.73(7)

Symmetry transformations used to generate equivalent atoms: *3 − *x*, − *y*, 2 − *z*, ^†^1 − *x*, 1 − *y*, 1 − *z*.

**Table 4:** SOD activity profiles of compounds (**1**)–(**11**) as assessed by the NBT assay.

Compound	Complex	Concentration (*μ*M) equivalent to 1U SOD

**(1)**	[Cu(2dmepda)(H_2_O)_3_]_2_	0.51
**(2)**	[Cu(3dmepda)(H_2_O)_3_]_2_	0.52
**(3)**	[Cu(2dmepda)(phen)(H_2_O]_2_ · 0.5phen	0.46
**(4)**	[Cu(2dmepda)(bipy)(EtOH)]_2_ · 2EtOH	0.88
**(5)**	[Cu(3dmepda)(phen)(H_2_O)]_2_	0.38
**(6)**	[Cu(3dmepda)(bipy)(H_2_O)]_2_ · 6H_2_O	0.37
**(7)**	[Mn(2dmepda)] · 1.5H_2_O	0.83
**(8)**	[Mn(3dmepda)] · H_2_O	0.77
**(9)**	[Mn(2dmepda)(phen)_2_]	0.89
**(10)**	[Mn_2_(2dmepda)_2_(bipy)_3_] · H_2_O	0.68
**(11)**	[Mn(3dmepda)(phen)_2_] · 7.25H_2_O	0.96

**Table 5:** Cancer chemotherapeutic potential of (**1**)–(**6**), phen, and the free copper salt was determined in HepG2 and A-498 cells, following continuous incubation for 96 hours in the concentration range of 0.1–500 *μ*M, using MTT assay. A graph of viability as % of solvent-treated control versus drug concentration was used to calculate IC_50_ values (*μ*M), (mean ± SEM; *n* = 5). Results are representative of three independent experiments (*n* = 3).

Complex	HepG2	A-498
	IC_50_(*μ*M) Mean ± S.E.M.	IC_50_(*μ*M) Mean ± S.E.M.

[Cu(2dmepda)(H_2_O)_3_]_2_ (**1**)	389.00 ± 20.00	110.00 ± 51.32
[Cu(3dmepda)(H_2_O)_3_]_2_ (**2**)	180.00 ± 50.35	95.00 ± 10.00
[Cu(2dmepda)(phen)(H_2_O)]_2_ · 0.5phen (**3**)	1.70 ± 0.08	1.55 ± 0.08
[Cu(2dmepda)(bipy)(EtOH)]_2_ · 2EtOH (**4**)	1.55 ± 2.31	9.50 ± 1.81
[Cu(3dmepda)(bipy)(H_2_O)]_2_ · 6H_2_O (**6**)	26.00 ± 6.05	15.50 ± 6.67
Phen	5.80 ± 0.31	4.1 ± 0.54
Cu salt {Cu(ClO_4_)_2_ · 6H_2_O}	> 1000.00 ± 1.00	973.30 ± 26.67
